# miRNA-143 expression is associated with inflammation and time of exposure to amniotic fluid in experimental gastroschisis

**DOI:** 10.1016/j.clinsp.2023.100311

**Published:** 2023-11-25

**Authors:** Ana Maria Bicudo Diniz, Igor José Nogueira Gualberto, Luiza Almeida Lima, Mucio Luiz de Assis Cirino, Rodrigo Kendi Murakami, Bella Luna Colombini Ishikiriama, Rodrigo Ruano, Luiz Fernando Ferraz da Silva, Daniela Tirapelli, Lourenço Sbragia

**Affiliations:** a Division of Pediatric Surgery and Anatomy, Department of Surgery and Anatomy, Faculdade de Medicina de Ribeirão Preto, Universidade de São Paulo (USP), Ribeirao Preto, SP, Brazil; b Faculdade de Medicina de Bauru, Department of Pediatric Dentistry, Orthodontics and Public Health, Faculdade de Odontologia de Bauru, Universidade de São Paulo (USP), Bauru, SP, Brazil; c Division of Maternal Fetal Medicine, Jackson Fetal Care, Department Obstetrics, Gynecology and Reproductive Sciences, Miller School of Medicine, University of Miami, Miami, Florida, USA; d Department of Pathology, Faculdade de Medicina da Universidade de São Paulo (FMUSP), São Paulo Death Verification Service (SVO), São Paulo, SP, Brazil

**Keywords:** Gastroschisis, Rat, mRNA-143, Inflammation, Amniotic fluid

## Abstract

**Objective:**

Gastroschisis (GS) is a congenital anomaly in the abdominal wall with the intestinal loops exiting laterally to the umbilicus. The contact of the loops with Amniotic Fluid (AF) causes an inflammatory process in the exposed part, leading to an extended hospital stay and an increased risk of morbidity due to alterations related to intestinal motility. The authors aimed to evaluate the time of exposure to the AF in the experimental GS and to search for potential biomarkers of intestinal inflammation by measuring microRNAs.

**Methods:**

Rat fetuses were divided into three groups: a) CONTROL, b) GS reared on day 18 (GS = 18), and c) GS reared on day 19.5 (GS = 19) (term = 22 days). On day 21.5, the fetuses were removed for biometric parameters and biochemical analyses: 1) Biometrics: Body and Intestinal Weight (BW, IW), and intestinal-body weight ratio (IW/BW); 2) Descriptive histopathology and 3) miR-143 quantification by real-time Polymerase Chain Reaction (PCR).

**Results:**

BW was higher in CONTROL than GS 18 and G19 (p < 0.05). IW, IW/BW, intestinal water, and mRNA-143 were higher in GS 18 and GS 19 than in CONTROL, and GS 18 was higher than GS 19 (p < 0.05). The average of the inflammation score from the intestinal wall with mucosal inflammation and intra-epithelial lymphocytes shows worst in GS 18 and GS 19 vs. CONTROL (p < 0.05).

**Conclusions:**

The tissue expression of mRNA-143 and the morphological changes in the intestine of GS worsened according to the time of exposure to AF, which could be a possible marker of fetal intestinal damage.

## Highlights

• miR-143 is related with more inflammation and edema of intestinal loops of gastroschisis rat model.
• Time of exposure of the amniotic fluid increases the intestinal damage.
• miR-143 is a possible marker of gut inflammation.

## Introduction

Gastroschisis (GS) is a congenital abdominal wall defect characterized by a small hole, usually located to the right of the umbilicus. It allows herniation and permanent exposure of the intestinal loops to Amniotic Fluid (AF) and its components during pregnancy.^1^

The permanent exposure of the loops to AF and its components causes alterations in the morphology and the histology of the intestinal wall, which leads to intestinal hypomotility and nutrient absorption deficiency.^2^ Hypomotility and intestinal absorptive deficiency, in turn, require prolonged parenteral nutrition and increase the possibility of postoperative complications, which increases morbidity, mortality, and the cost of medical-hospital treatment.^3^

GS, which occurs in 1:5,000 to 1:10,000 live births, affects more boys than girls in an approximate ratio of 2:1 and more fetuses of mothers aged under 20 years of low socioeconomic status, low grade of schooling, associated with an obstetric history of abortion and a short time interval between menarche and the first pregnancy.^4^^,^^5^ The mortality rate of GS varies from 4 % to 22 %, but can reach 28 % when associated with intestinal atresia or perforation and 100 % when midgut volvulus occurs.^6^

The immaturity of the GS myenteric plexus reduced the expression of α-internexin and peripherin (neurofilaments proteins) in the neuron plexus, two proteins necessary in the formation of the cytoskeleton and neuronal maturation. Furthermore, a reduction in synaptophysin expression, an integral membrane protein involved in the conduction of synaptic vesicles, was identified. Synaptophysin is considered a signal of specification of neuronal function and maturation of nerve cells in prenatal life.^7^ This maturity was corroborated concerning the time of exposure to the Amniotic Fluid (AF) because the longer the time of exposure of the loop to the AF, the greater the neuronal immaturity.^8^ MicroRNAs (miRNA) are a group of small RNA molecules, which are single-stranded, consisting of approximately 19 to 25 nucleotides that act as regulators of gene expression, acting at the post-transcriptional level by blocking protein synthesis or induction of mRNA degradation.^9^ Since microRNA is involved in mRNA expression and, consequently, in protein transcription, it would allow an understanding of the inflammatory process present in GS. Tissue from human GS neonates demonstrated increased miRNA 143 & 145 expressions in intestinal smooth muscle cells, consequently promoting the activation of Transforming Growth Factor-Beta 3. This activation promotes changes in intestinal smooth cell function and corroborates with the intestinal dysfunction of the disease.^10^ Therefore, the present study's objective was to evaluate the expression of miRNA-143 involved with inflammation according to the exposure time of the intestinal loops in experimental GS.

## Material & methods

All procedures followed the guidelines of the National Council for the Control of Animal Experimentation (CONCEA, Brazil) and were approved by the Local Institutional Animal Care and Use Committee of Ribeirao Preto Medical School, University of Sao Paulo (CEUA protocol n° 034/2021).

### Animal

Male and female Sprague Dawley rats weighing around 250g were subjected to mating. The couple was kept together for one night. The next day, the female's genital region was examined for vaginal sperm staining. The presence of a vaginal spot configured mating and was considered day zero of pregnancy (term = 22 days). The animals were kept in cages with food and water offered ad libitum, under controlled conditions of light (12 h of light/12 h of dark), temperature (average of 23 °C), and relative humidity close to 55 %.

### Constitution of the groups

Three groups were studied: a) Control: sibling fetuses not submitted to surgery; Gastroschisis 18 (GS 18), fetuses undergoing surgery to create a GS at 18 days of gestational age and Gastroschisis 19 (GS 19), fetuses undergoing surgery to make a GS at 19 days of gestational age.

### Surgery

Surgical procedures occurred in the morning, with a gestational age of 18.5 days. After acclimatization in the laboratory, the pregnant rats were submitted to general anesthesia with intramuscular injection of ketamine base – 50 mg/mL (Ketamina® ‒ Pfizer do Brasil) associated with xylazine 10 mg/mL (Rompum® ‒ Bayer do Brasil Ltda) in the dose of 0.6 mL per animal intramuscularly applied to the lateral muscles of the thigh with an insulin syringe and 20G needle. This anesthetic composition (180 mg/kg of ketamine and 1.25 mg/kg of xylazine) maintains the animal under deep anesthesia for three hours, and the postoperative period is painless between 6 and 12 h. The abdomen was subjected to hair shaving with an electric clipper, cautious not to injure the nipples. The animals were placed on a plate heated by electrical resistance, brand Harvard Apparatus®, and previously regulated at a temperature of 38 °C. After asepsis with chlorhexidine (Chlorohex®) aqueous solution and placement of sterile drapes, the animal underwent median laparotomy in two planes (skin and aponeurosis/peritoneum). Then, the bicornuate uterus was gently exposed and protected with sterile gauze soaked in physiological saline solution heated to 38 °C. Fetuses were counted from the proximal region to the distal part of the uterine cervix, starting with the right horn. Each horn's first and last fetus was not used in the experiment due to the risk of miscarriage related to handling these animals. In general, Sprague-Dawley rats conceive 8 to 12 pups per pregnancy. The number of fetuses submitted to GS was 3 to 4 per pregnant rat at 18.5 and 19.5 days. The surgical creation of the GS was performed according to Correia-Pinto et al.^11^ Using a surgical loupe of 2,5 × magnification, a purse-string suture with 6‒0-gauge Prolene® thread was performed on the uterine wall. Then, the uterus was opened into its two layers, muscle, and amniotic membrane. The fetus was exposed through the uterine incision up to the level of insertion of the umbilical cord and kept with the upper abdomen and chest inside the mother's uterus. Then, the right and left lower limbs were partially removed to facilitate exposure of the abdominal region to be incised. This technical maneuver decreases the risk of injury to the umbilical vessels during the GS procedure. The fetal abdominal cavity was opened through a right paraumbilical laparotomy incision, with an approximate length of 3 mm, taking care not to damage the umbilical vessels and the liver. The intestinal loops were quickly exposed by gently compressing the fetal abdomen with flexible sterilized cotton swabs. After creating the GS, the fetus was carefully replaced in the uterine cavity, and the uterus was closed using the previously served purse-string suture. The subsequent fetus called the control, was neither submitted to surgery nor removed from the uterus. During the procedure, the maternal uterus and the exposed portion of the fetus were kept warm with saline solution (NaCl 0.9 %) at 38 °C, dripped with a 5 mL syringe. At the end of the procedure on the available fetuses, the abdominal wall of the mother rat was closed in 2 planes, using 4-0 Mononylon® thread with continuous suture. The rats were recovered with inhaled oxygen in a properly adapted mask at 1 L/min until they were fully awake and moving without problems. The postoperative period occurred in acrylic cages, in individual pens, with food and water offered ad libitum.

### Harvest

Pregnant rats were anesthetized again and underwent cesarean section delivery on day 21.5 of gestational age, and their fetuses were harvested and sacrificed. Measurements of neonatal body and intestinal weight (from the duodenum to the rectum) were performed immediately after collection. The intestine was immediately fixed in a suitable solution for later histological analysis. Fetuses from groups destined for molecular analysis were immediately frozen in liquid nitrogen.

### Biometrics parameters evaluation

Body Weight (BW), Intestinal Weight (IW), and the IW/BW mathematical ratio were measured (%) to exclude the BW variable from the IW assessment.

### Intestinal wet-dry ratio

The intestinal tissue was placed on the weighed tin foil. Firstly, the intestinal tissues were considered before drying in an oven at 60 °C. Secondly, they were taken out after 24 h, the dry weight was obtained. W/D = (Wet Weight ‒ paper weight) / (Dry weight ‒ paper weight) (n = 6 samples for each group).^12^

### Histological evaluation

The intestine was harvested from the ileum, the part most exposed to LA. The frames will be fixed in fresh 4 % paraformaldehyde solution and stained with Hematoxylin/Eosin (H&E) for histological identification. Histological sections were made in the transverse direction, with a thickness of 5 µm for histometric measurements using the Image Pro Plus® program (Rockville, MD, USA).

The inflammation was assessed, by two different experts, in samples collected from CONTROL (n = 7), GS 18 (n = 5), and GS 19 (n = 5) neonate rats, using a semi-quantitative scoring system to determine the extent of inflammatory cell presence in the mucosa, as well as intraepithelial lymphocytes.

All evaluations were analyzed using at least 10 high-power fields per case. Mucosal inflammation was semi-quantified using four categories (0 to 3), representing the following levels of mucosal infiltration with different inflammatory cells: 0 = minimal (mostly mononuclear cells), 1 = mild (increased number, most mononuclear cells, some polymorphonuclear cells), 2 = moderate (large number, with both mononuclear and polymorphonuclear cells), and 3 = severe (largest number with predominantly polymorphonuclear cells).

The score 0 (minimal) was considered the normal bowel, as it usually keeps minimal mucosal inflammation. The presence of intraepithelial lymphocytes was semi-quantified using four categories (0‒3), indicating the following levels: 0 = absent, 1 = minimal (up to 3 intraepithelial lymphocytes/high-power field), 2 = mild/moderate (3‒10 lymphocytes/high-power field), and 3 = severe (> 10 lymphocytes/high-power field).^13^

### MicroRNA selection

The miR-143 microRNA was selected through a literature review and an in-silico search with data crossing in target prediction platforms. (MiRDB, TargetScan, DianaTools, GeneCards).

### RNA isolation and real-time polymerase chain reaction

Total RNA was extracted using a Trizol reagent (Applied Biosystems, Foster City, CA, USA) following the manufacturer's instructions. In preparation for the real-time Polymerase Chain Reaction (PCR), reverse transcription of RNA samples was performed using the High-Capacity cDNA kit (Applied Biosystems). The cDNA was amplified with Quantitative real-time Polymerase Chain Reaction (Q-PCR) using TaqMan Master Mix (Life Technologies, Carlsbad, CA, USA) to respond to microRNAs. The specific probes and assay ID for miR-143 was 000466 from qPCR Taqman™ (Life Technologies). The U6 gene was used as an endogenous control (housekeeping) for the reaction of the microRNA. The PCR conditions included pre-heating at 50 °C for two minutes, denaturation at 95 °C for ten minutes, and 50 cycles of amplification and quantification (15 s at 95 °C and one minute at 60 °C). The 7500 Sequence Detection System apparatus (Applied Biosystems) duplicated and analyzed all reactions. The data were analyzed using ABI-7500 SDS software. Dissociation curves were performed (melting curves) after amplification by RQ-PCR. The samples that showed dissociation curves with different temperatures or more than one point of dissociation in the same sample were discarded and repeated for miRNA analysis (n = 6 per group).

### Statistical analysis

To describe the sample profile according to the various variables under study, frequency tables were created for categorical variables and descriptive statistics (with measures of position and dispersion) for continuous variables. Data were evaluated using the Kolmogorov–Smirnov test to assess evidence that they could have a normal distribution. Analysis of Variance (ANOVA) and Tukey's post hoc test for repeated measures was used to compare variables between groups and gestational ages. Statistical analysis was performed using the Kruskal–Wallis tests and Dunns and Mann–Whitney multiple comparison post-test to evaluate miRNA expression. The GraphPad Prism version 9.2 for Windows program (GraphPad Software, San Diego – California, USA) was used. Statistical significance was considered when p-values < 0.05.

## Results

A total of 20 pregnant rats were submitted to the GS creation, and one died of anesthetic complications. Of the remaining 19 rats, 37 fetuses were collected as controls, and 91 GS were performed. The survival rate in GS 18 was 42 % (21/50), and GS 19 was 51 % (22/41). The overall survival rate of both GS was 46 % (42/91).

### Biometric parameters

Body Weight (BW): CONTROL = 4.703 (± 0.429), GS 18 = 4.175 (± 0.557), and GS 19 = 4.123 (± 0.412). There was a difference between CONTROL and GS 18 and GS 19 (p < 0.005). Intestinal Weight (IW): CONTROL = 0.138 (± 0.028), GS 18 = 0.178 (± 0.045), and GS 19 = 0.135 (± 0.038). There were differences among GS 18, GS 19, and CONTROL (p < 0.005). Intestinal to Body Ratio (IW/BW): CONTROL = 0.029 (± 0.005), GS 18 = 0.042 (± 0.008), and GS 19 = 0.034 (± 0.008). There were differences among GS 18, GS 19, and CONTROL (p < 0.005).

**Fig. 1:**
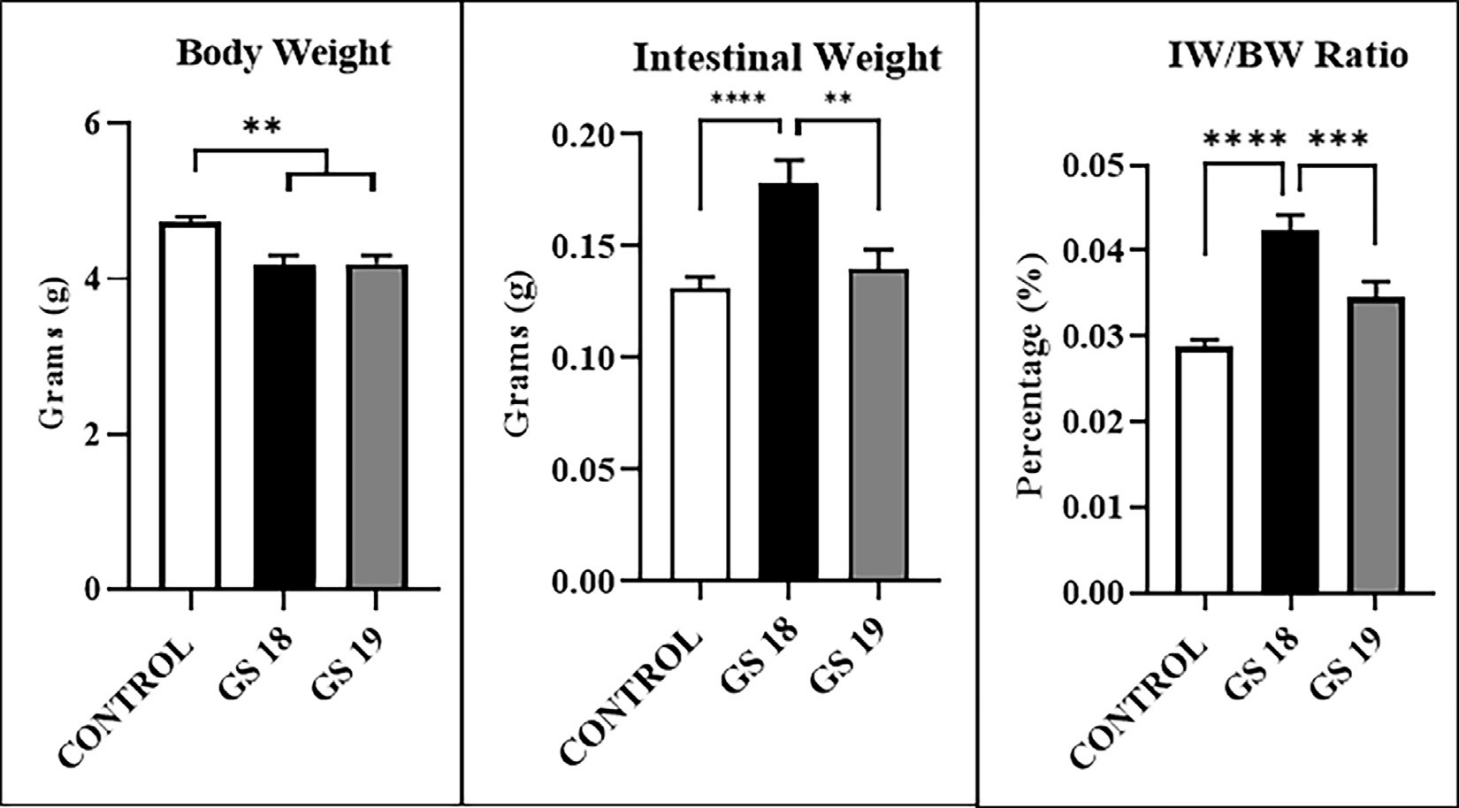
Represents the biometric parameters and comparisons among the groups with (SEM).

The biometric parameter results are in Table 1 and Fig. 1.

**Table 1:** Morphometric results of gastroschisis submitted to two different gestational ages.

	CONTROL (n = 37)	GS 18 (n = 21)	GS 19 (n = 22)	p
Body weight (BW) (g)	4.703 (± 0.429)	4.175 (± 0.557)	4.123 (± 0.412)	< 0.005^a^
Intestine weight (IW) (g)	0.138 (± 0.028)	0.178 (± 0.045)	0.135 (± 0.038)	< 0.0005^b^
Intestine to body ratio (IW/BW) (%)	0.029 (± 0.005)	0.042 (± 0.008)	0.034 (± 0.008)	< 0.0005^c^

BW: ^a^ CONTROL vs. GS 18 and GS 19; IW: ^b^ CONTROL vs. GS 18 and GS 18 vs. GS 19^c^ (p < 0.0001); IW/BW: ^b^ CONTROL vs. GS 18 and GS 18 vs GS 19^c^ (p < 0.0001).

### Intestinal wet-dry ratio

**Fig. 2:**
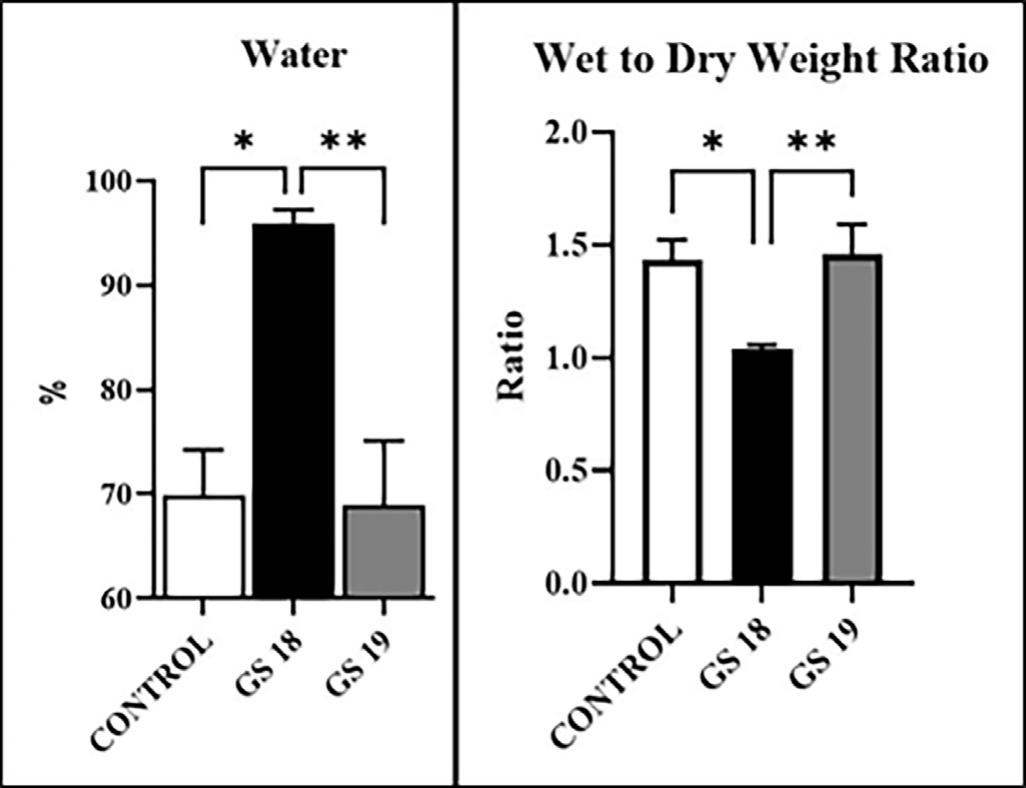
Represents the amount of intestinal water after two different times of exposure of amniotic fluid after creation of gastroschisis in rat model. * p < 0.05 GS 18 × Control, and ** p < 0.005 and GS 18 × GS 19.

The results of wet-dry is shown in Fig. 2.

### Macroscopic and histological findings

**Fig. 3:**
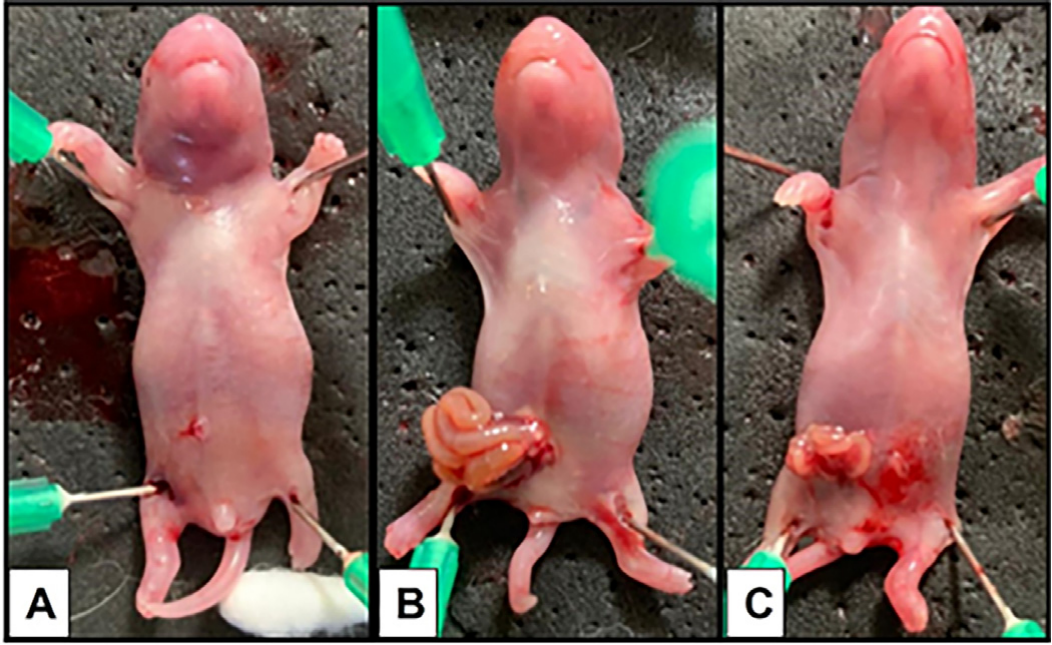
Represents the macroscopic view of gastrochisis at different gestational ages. (A) Neonate control; (B) Neonate with GS 18 shows the intestinal loops with edema and inflammatory aspect and (C) Neonate with GS 19 shows less edema and a decrease of inflammatory.

**Fig. 4:**
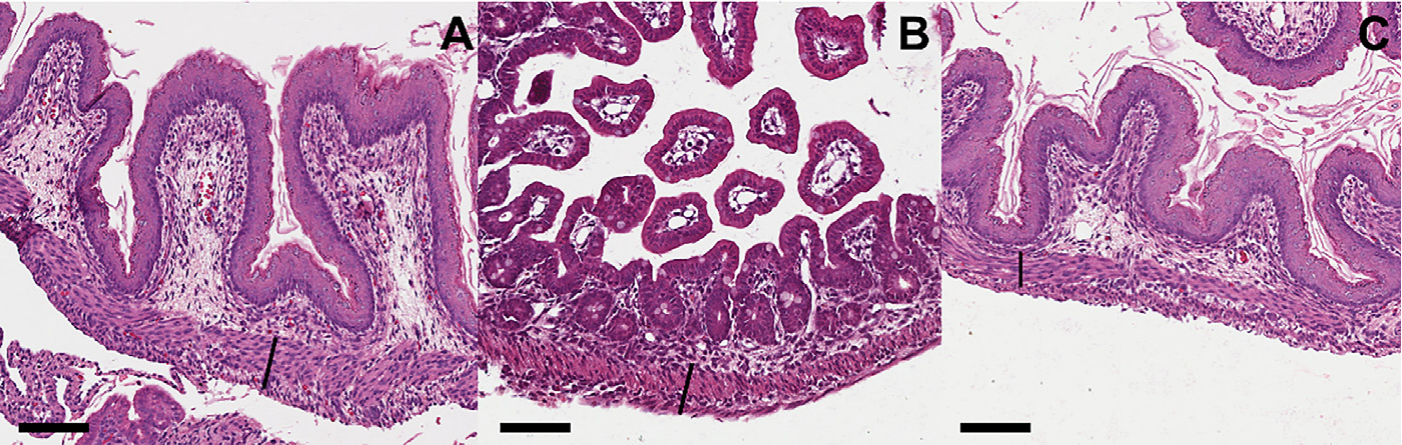
Represents the histological findings of the gastroschisis villus indifferent gestational ages. Cross sections of intestinal villi obtained from GS 18 (A), GS 19 (B) and Controls (C). Note the increased thickness of the muscle layer of the bowel of the fetuses with gastroschisis. Scale bar = 100 µm.

**Fig. 5:**
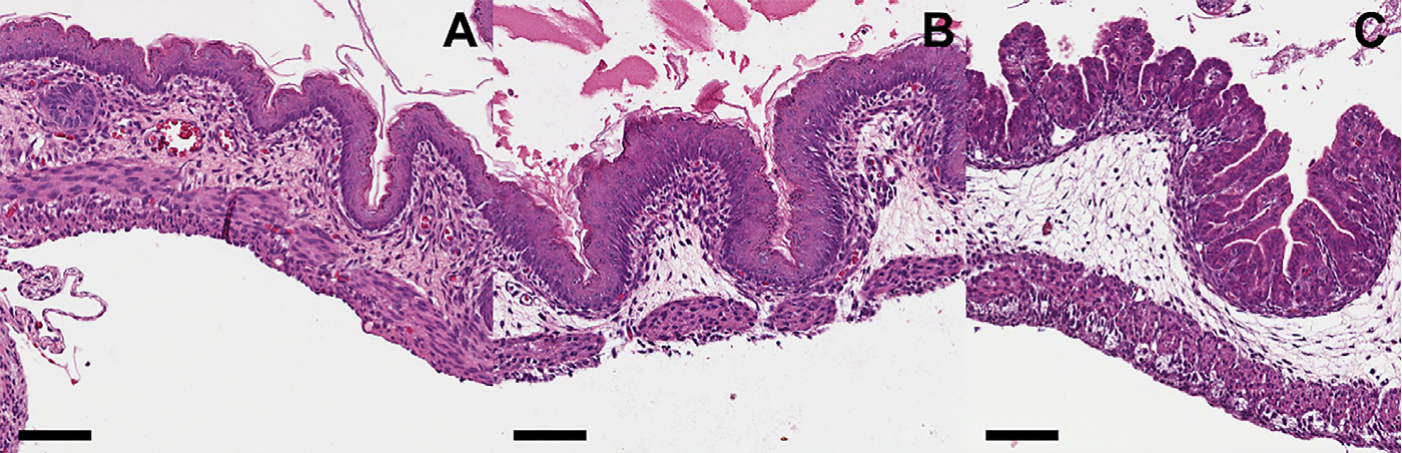
Represents the inflammatory findings of the gastroschisis villus indifferent gestational ages. Cross sections of intestinal villi obtained from GS 18 (A), GS 19 (B) and Controls (C). Note the increased number of inflammatory cells in the mucosa of the bowel of the fetuses with gastroschisis groups. Scale bar = 100 micrometer.

The Fig. 3 shows the macroscopic view and the Table 2 with the Fig. 4 the microscopic findings and microphotographic view (Fig. 5).

**Table 2:** Represents the average of the inflammation score from intestinal wall.

	Mucosal inflammation	Intra-epithelial lymphocytes
CONTROL (n = 7)	0.5	0.2*
GS 18 (n = 5)	1.5	1.2
GS 19 (n = 5)	1.2	1.0

⁎ p < 0.05 Control vs. GS 18 and GS 19.

### MicroRNA

**Fig. 6:**
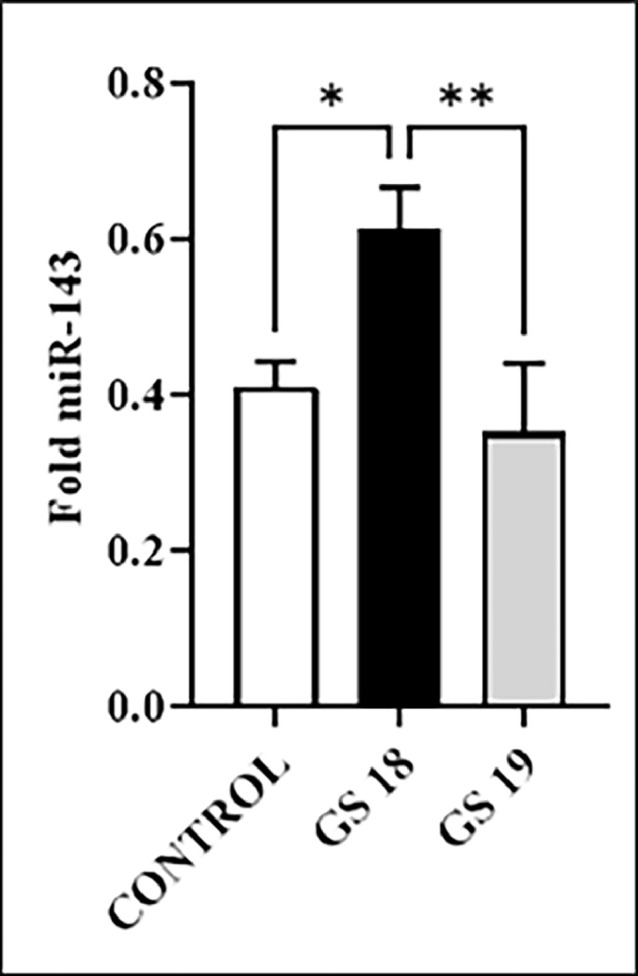
Shows the amount of miR-143 in the intestinal tissue of fetal rats.

The results of mRNA-143 is shown in Fig. 6.

## Discussion

GS is a defect in the anterior abdominal wall, lateral to the umbilicus, through which the abdominal viscera project into the uterine cavity. The contact of the intestine with some AF components causes an inflammatory process and muscular and nervous alterations in the intestinal wall that cause intestinal dysmotility, which lead to an extended hospital stay.^14^ Due to this, there is a high economic cost resulting from the treatment, whose average cost per hospitalization is US$ 114,230.00 per patient.^15^ Although GS survival is high, around 95 %, the association of the defect with atresia, perforation, intestinal volvulus, and necrotizing enterocolitis is related to a more significant number of complications and a greater risk of neonatal mortality.^16^

The diagnosis is made during prenatal morphological ultrasound. The absence or delay in prenatal diagnosis is a factor in more significant morbidity and mortality.^17^ Therefore, searching for a prenatal biochemical marker to predict neonatal prognosis could provide the most appropriate management of babies with GS and reduce morbidity.

The surgical rat model of GS has the advantage of being inexpensive, having a short gestation period with many fetuses per pregnancy, and the surgical procedure has a reasonable survival rate (∼90 %). Furthermore, intestinal tissue damage occurring in the last trimester of human gestation is well mimicked in the last three days of rat gestation.^11^

In this experiment, the authors chose to evaluate the effects of Amniotic Fluid (AF) in the intestine of the GS at two gestational ages (18 and 19 days). The surgery of the GS 18 group was performed one day before the GS 19 group so that the GS 18 group had a longer exposure time to AF.

The authors observed greater BW in control fetuses than in GS 18 and GS 19 fetuses (p < 0.05), with greater IW in GS 18 fetuses than in GS 19 and control fetuses, respectively (p < 0.05 and p < 0.005). In addition, the IW/BW Ratio demonstrated that the IW component of the total weight of GS 18 is greater than that of GS 19 (p < 0.005), which suggests that the greater weight of the GS 18 group is due to the inflammatory process, edema, the thickening of the intestinal wall and formation of a fibrous lining over this tissue, and not to a gain in body weight distributed in the fetus.^18^

The most common descriptive findings of the histology of the intestinal loops in GS in humans and experimental in sheep refer to the increase in the thickness of the intestinal muscle layers that double or become up to six times more significant with edema between the layers and increases of the serous layer with the presence of fibrosis associated with mild infiltration by polymorphonuclear leukocytes and mononuclear cells.^19^

Evaluation of experimental GS in sheep showed that serosa damage is related to ischemia and exposure to AF.^20^ The authors compared loops exposed and not exposed to AF, and with and without constriction, and found more fibrous shell, dilation of mesenteric and lymphatic veins, and blunting of mucous villi only in association with loops in contact with exposure to amniotic fluid.^20^

In the same model, the lesion caused in the circular and longitudinal muscles becomes more evident closer to the term. It can be reversed if the intestinal loops are protected from exposure to AF.^21^ The thickening of the musculature a nd low motility can be explained by the increase in collagen deposits, where in the beginning, there is hyperplasia of the intestinal musculature, and at the end, there is hypertrophy and a decrease in absorption.^22^

Urine and meconium components associated with ischemia of the fetal intestinal loops in GS have an inflammatory effect on the serosa.^23^^,^^24^ The capacity for a quick turnover of loops exposed to AF by ex-changing AF or increasing diuresis may have a protective effect by decreasing edema and loop thickness and, therefore, decreasing morbidity.^25^

The authors found that GS 18 and GS 19 had more significant inflammation than the control, with more mucosal inflammation and intra-epithelial lymphocytes for the field in GS 18 but without statistical difference with GS 19. These findings agree with the other previously described authors.

The wet-dry ratio intestinal analysis method can be used to evaluate the damage to the intestinal mucosa in rats by assessing the amount of fluid accumulated in the intestinal tissue.^13^ The formation of intestinal edema, inflammation, and ileus are commonly found in various inflammations and cirrhosis and are common in GS. Excessive intraoperative crystalloid fluid administration during surgery results in intestinal edema and postoperative ileus.^26^ In addition, smooth muscle endothelial dysfunction contributes to interstitial edema.^27^ In previous work, the authors found that the amount of Protein per Weight of Intestine (IP/IW) in GS was higher compared to the controls. In addition, the treatment with prenatal steroids seemed to decrease the amount of water in the tissue and the treatment with higher doses of the same steroid was responsible for further decreasing the wall thickness, especially in the mucosa leading to secondarily increasing the glucose absorption by promoting the maturation of maltases and lactases.^28^^,^^29^ In this way, a more significant component of edema was observed in GS 18, a result that corroborates with the morphometric findings regarding the accumulation of water in the intestine of the GS with the most prolonged exposure to AF. The more pronounced inflammatory process at the serosa's level with collagen deposits and edema is a primordial component of intestinal loop dysmotility. Once intestinal length is recovered after edema, the intestine will return to normal motility.^30^^,^^31^

In a model of Superior Mesenteric Vein (SMV) obstruction in adult rats to study the component of intestinal wall edema on motility and early absorption of food, it was reported that edema may promote changes in the genes involved in the regulation of the actin cytoskeleton by decreasing the expression of myosin light chain kinase mRNA.^32^ The edema also has been suggested to inhibit smooth muscle cell proliferation, promoting apoptosis and upregulation of Transforming Growth Factor beta (TGF-β) transcription, stimulating collagen deposition.^33^

Blood miR-143 has been identified as a potential biomarker for diagnosing tumors such as bladder cancer and acute myeloid leukemia.^34^^,^^35^ There is little report related to the use of miR-143 for fetal diagnosis except for its placental tissue dosage, where high levels of miR-21 expression and low levels of miR-143 expression predict the risk of fetal macrosomia.^36^ miR-143 is related to several processes linked to the pathophysiology of GS by regulating smooth muscle contraction, intestinal angiogenesis, and cellular response to DNA damage.^37^ Overexpression of miR-143 leads to increased intestinal inflammation,^38^ such as the genes of the Mitogen‑Activated Protein Kinase (MAPK) family,^39^ Intercellular Adhesion Molecule 1 (I-CAM 1) and Nitric Oxide Synthase 3 (NOS 3),^40^ and TGF-β3 which is more involved in intestinal motility dysfunction.^10^

The present results demonstrate a higher expression of miR-143 in GS 18 compared to the control group and GS 19. The hypothesis is that the miRNA amplification may occur due to an intestinal inflammatory process leading to cell damage with more significant duration and intensity. The present hypothesis is that this inflammatory process may be possibly due to some component present in AF, a fact that was not observed sufficiently in GS 19.

This study has some limitations including the fact that the authors tried to quantify the miRNA in the fetal blood and amniotic fluid, which needs still to obtain amplification. Another probe could be used to amplify this miRNA in the future. Furthermore, the authors did not focus on the findings of these alterations with the quantification of TGF-β3 due to the small amount of material for molecular biology.

Finally, the authors can conclude that there is a relationship between the time of exposure to amniotic fluid and the level of intestinal edema and inflammation suggested by the increased tissue expression of miR-143. Future studies are still necessary to confirm the present hypothesis in larger animals and to investigate the miR-143 role as a possible candidate biomarker for the severity of GS inflammation related to TGF-β3 pathway and the disease morbidity.

## CRediT authorship contribution statement

**Ana Maria Bicudo Diniz:** Investigation. **Igor José Nogueira Gualberto:** Investigation. **Luiza Almeida Lima:** Data curation. **Mucio Luiz de Assis Cirino:** Methodology. **Rodrigo Kendi Murakami:** Methodology. **Bella Luna Colombini Ishikiriama:** Methodology. **Rodrigo Ruano:** Writing – review & editing. **Luiz Fernando Ferraz da Silva:** Methodology, Supervision. **Daniela Tirapelli:** Supervision. **Lourenço Sbragia:** Writing – review & editing.

## Declaration of Competing Interest

The authors declare no conflicts of interest.
